# Implementation of the I-PASS Handover Framework in an Internal Medicine Unit at a Sudanese Teaching Hospital: An Exploratory Pre-Post Audit

**DOI:** 10.7759/cureus.109814

**Published:** 2026-05-28

**Authors:** Rudaina I Osman Ahmed, Isra M Mohamed, Omer K Abdelmoneim, Mohammedelfatih A Mohammedelamin

**Affiliations:** 1 Internal Medicine, Napta College, Khartoum, SDN; 2 Internal Medicine, The National Ribat University, Khartoum, SDN

**Keywords:** adherence, communication, handoff, handover, implementation, i pass, low resource settings, sudan

## Abstract

Purpose: This pre-post implementation audit aimed to evaluate adherence to the I-PASS (illness severity, patient summary, action list, situation awareness and contingency planning, and synthesis by receiver) handoff framework before and after an educational intervention in the Internal Medicine Department at Ribat Teaching Hospital.

Background: Effective patient handover is critical for continuity and quality of care, and miscommunication during transitions is a major contributor to preventable harm. While structured tools such as I-PASS have improved handoff quality in high‑resource settings, evidence from low‑resource environments, including Sudan, remains limited. Teaching hospitals in Sudan face overcrowding, workforce shortages, and fragmented information systems, which increase the risk of communication errors.

Materials and methods: This exploratory quasi-experimental pre-post implementation audit was conducted in one Internal Medicine Unit at Ribat Teaching Hospital, Khartoum, Sudan. All handoffs for inpatients requiring close monitoring during the study period were eligible. Two cycles of observation were completed. In each cycle, adherence to the five I‑PASS elements was recorded using a checklist during live verbal handoffs. Between cycles, a structured educational program on I‑PASS was delivered to unit staff. Data were analyzed descriptively, and Fisher’s exact test was used to compare adherence proportions between cycles.

Results: A total of 105 handoffs were observed (55 in cycle one and 50 in cycle two). In cycle one, adherence varied across I‑PASS elements: illness severity 22/55 (40%), patient summary 37/55 (67%), action plan 55/55 (100%), situational awareness 20/55 (36%), and synthesis by receiver 37/55 (67%). After the educational implementation, adherence improved for most elements: illness severity 43/50 (86%; p<0.001), patient summary 44/50 (88%; p=0.019), action plan 50/50 (100%; p=1.000), situational awareness 42/50 (84%; p<0.001), and synthesis by receiver 48/50 (96%; p<0.001).

Conclusion: The I‑PASS framework was feasible to implement in a low‑resource internal medicine unit and was associated with improved short‑term adherence to structured handoff elements after targeted education. The study did not measure adverse events or error rates; therefore, no conclusions can be drawn about the impact of I‑PASS on patient safety. Longer‑term, multi‑unit evaluations incorporating safety outcomes and potential confounders are warranted.

## Introduction

A handover is the process in which vital patient information is transferred between healthcare providers or teams, ensuring continuity and safety in patient care. Effective handovers are crucial for maintaining treatment consistency and minimizing risks. The structure of a handover consists of both content and method; content can be organized using tools such as templates, mnemonics, and checklists, or it may remain unstructured, while the method refers to the mode of communication, whether verbal, written, or recorded [1,2].

Miscommunication during handovers is a leading cause of medical errors in hospitals, significantly impacting patient outcomes and caregiver confidence [3,4]. Data from the Centers for Disease Control and Prevention and medical literature highlight that medical errors are the third leading cause of death in the United States [5]. Recognizing this, the Accreditation Council for Graduate Medical Education and the Joint Commission have emphasized the need for accurate and efficient transitions of care as a crucial strategy for enhancing patient safety and medical education [3]. Additionally, a study by Kitch et al. in 2008 revealed that ineffective handovers between healthcare workers led to 59% of residents reporting that one or more patients suffered from complications, of which 12% of them reported significant harm [6]. The World Health Organization (WHO) identifies patient handoffs as a fundamental aspect of quality healthcare, underscoring the necessity of effective information and responsibility transfer among healthcare professionals [5].

Several structured approaches have been introduced to improve the quality and consistency of clinical handovers. Commonly used communication tools include SBAR (situation, background, assessment, recommendation); SOAP (subjective, objective, assessment, plan); and I-PASS (illness severity, patient summary, action list, situation awareness and contingency planning, and synthesis by receiver) [7]. Among these, I-PASS was specifically developed to promote more comprehensive and standardized patient handoffs and to address limitations observed with simpler communication frameworks such as SBAR [8,9].

The implementation of structured handover processes has demonstrated significant improvements in patient safety. Research by Starmer et al. found that standardizing handovers using the I-PASS program led to a 23% reduction in medical errors and a 30% decrease in preventable adverse events across nine pediatric hospitals [10].

Despite the benefits of structured handovers, implementing these processes remains challenging, particularly in resource-limited settings. A major obstacle is achieving compliance and engagement from healthcare providers, as behavioral changes in communication practices require consistent reinforcement and institutional support [11-13].

Effective handoff communication is critical for ensuring patient safety in clinical settings, particularly in resource-limited environments. While structured communication frameworks such as I-PASS have demonstrated success in high-resource countries, their applicability and effectiveness in low-resource settings, such as Sudan, remain underexplored. In Sudan, the healthcare system faces challenges such as overcrowded hospitals, limited staffing, and a lack of standardization in handoff practices, which can contribute to communication errors. This study seeks to evaluate the implementation of the I-PASS framework in the Internal Medicine Department at Ribat Teaching Hospital, a leading teaching hospital in Sudan. By examining the I-PASS framework in this specific setting, we aim to contribute to the understanding of how structured handoff communication can improve patient safety in resource-limited environments. This is particularly significant given the scarcity of research on handoff practices within Sudanese healthcare institutions and the broader context of low-resource countries [14].

The health system in Sudan faces significant challenges, including unclear policies and implementation mechanisms, weak communication between health care sectors, a poorly functioning referral system, a shortage of postgraduate training, and a shortage of logistics supplies [15]. There was a noticeable shortfall in research regarding handoff in Sudan, making it difficult to have a clear image or accurate data due to the gap [16]. Furthermore, in comparison with existing literature, which mainly focuses on high-income countries that have a steady workflow, electronic health records, and well-established training programs, there is a significant lack of studies in low-income countries with increased workflow and a shortage of well-trained health workers. The benefits of implementing I-PASS as a handover tool in developing countries remain unclear, resulting in a significant gap in worldwide patients’ safety efforts [16]. Teaching hospitals in Sudan play a critical role in delivering healthcare services. However, like many resource-limited settings, these institutions face significant challenges in maintaining effective and standardized communication during patient handovers. These challenges consist of communication issues, such as language barriers or incomplete information, and patient-related issues, like complicated cases and frequent transfers. Increased workload and fatigability of the health care worker also pose a significant challenge, and the fact that there has been limited research on structured handover practices in Sudan makes it difficult to assess and address gaps in communication strategies [16].

By auditing current handover practices and assessing adherence to the I-PASS handoff framework, which consists of five components: illness severity, patient summary, action list, situational awareness, contingency planning, and synthesis by receiver, this study seeks to provide data-driven insights that can contribute to enhancing staff communication in Sudan's healthcare system.

## Materials and methods

Study design

After approval by the Internal Medicine Department, an exploratory pre-post implementation audit (quasi‑experimental study without a control group) was conducted to evaluate adherence to a customized handover protocol based on the I‑PASS framework (Appendix 1). The audit consisted of two cycles: cycle one assessed baseline handoff practices, followed by educational sessions for the internal medicine medical team; cycle two evaluated adherence after the educational implementation.

Ethical considerations

Verbal consent was obtained from all healthcare providers involved in the handoff process prior to observation. The Internal Medicine Department’s Institutional Review Board (IRB) reviewed and approved the project as a minimal‑risk quality improvement audit (University of Khartoum, Soba University Hospital, approval number SUH341769R12). In line with institutional policy for quality improvement activities that observe routine clinical practice and deliver department‑level education, the IRB determined that written consent was not required because no experimental clinical interventions were introduced and no patient‑identifiable data were collected.​ All data were anonymized, and the Google Form (Google LLC, Mountain View, CA, USA) used for data collection did not capture any personal identifiers for patients or staff. Electronic data were stored on password‑protected, encrypted devices accessible only to authorized research personnel, in accordance with institutional data protection guidelines. 

Study area

There are four units in the Internal Medicine Department at Ribat Teaching Hospital, Khartoum, Sudan. This audit was implemented in one of these units to ensure a focused and manageable implementation of the I-PASS handoff program, allowing for thorough evaluation and refinement before potential expansion to other units. The average number of admissions in this unit is around 28 patients per month.

The unit consists of one consultant, three registrars, four medical officers, and 15 house officers. Daily handoffs were conducted as structured verbal meetings at the nurses’ station or team room, typically at shift change, with house officers presenting patients and medical officers or registrars available to supervise and clarify plans.

Population and sampling

This study employed a convenience sampling approach, where all adult inpatients requiring close monitoring in the Internal Medicine Department of the Ribat Teaching Hospital during the study period were included in the analysis. This method was chosen due to logistical constraints, including time limitations and the dynamic nature of hospital admissions. Patients who were admitted during the study period and who required monitoring under the care of internal medicine were eligible for inclusion. Exclusion criteria were applied to patients discharged directly from the Emergency Department without being admitted to the study unit, or those who were transferred to another unit or hospital before completing their care, as their handoff process would not be fully documented. The sample size was determined based on available patient admissions during the observation period.

This sampling approach was justified given the aim of evaluating the real-world applicability and effectiveness of the I-PASS handoff protocol in a clinical setting where immediate patient care decisions are critical [17]. Handoffs were usually delivered by house officers, with medical officers and registrars present to review and modify plans when needed; this supervision model did not change between cycles.

Observers and measurement

Three observers (two registrars and one medical officer) from the Internal Medicine Department audit team attended handoff meetings in real time. They used a structured Google Form checklist to document whether each of the five I‑PASS components: illness severity, patient summary, action plan, situational awareness and contingency planning, and synthesis by receiver was mentioned during the verbal handoff. For the purposes of this audit, “documented” indicates that the observer recorded the presence of the element on the checklist during the verbal exchange; the quality, depth, and accuracy of each element were not graded. Before data collection, observers received training that included a review of the I‑PASS framework, use of the checklist, and practice with mock handoff video recordings. During a brief pilot phase, two observers rated a sample of handoffs simultaneously and discussed discrepancies to harmonize interpretation; no formal interrater reliability coefficients were calculated.

I-PASS sheet

The I-PASS Patient Safety Institute defines I-PASS as a structured communication tool consisting of written and verbal components [7]. The audit standard was set at 85% by consensus, considering the high workload and human factors in the hospital. Achieving 85% was seen as a realistic target, as it represents meaningful progress and improvement, even if not perfect.

Data collection

The study, conducted from 17/01/2023 to 28/03/2023, meticulously analyzed 105 handoffs to evaluate the efficacy of the I-PASS protocol.

I-PASS checklist

The tool used to assess adherence to the I-PASS handoff protocol was a customized Google Form checklist designed to track each of the five components of the I-PASS framework: illness severity, patient summary, action plan, situational awareness, and synthesis by receiver. Each component was assessed as either documented or not documented, based on its inclusion during handoff discussions.

Format

The checklist was structured so that each of the I-PASS components was listed as a separate unit, with checkboxes next to each one. The form was designed to allow observers to mark the presence or absence of each component during the handoff.

Validation

The checklist was validated through expert review by senior faculty members from the Internal Medicine Department to ensure that it accurately captured all relevant elements of the I-PASS framework. Additionally, a pilot phase was conducted in which the checklist was used in real clinical settings to identify any issues with clarity or usability. Feedback from the pilot phase was incorporated into the final version of the checklist to ensure its practicality and ease of use. Cycle one, which was a two-week baseline period from 17/01/2023 to 31/01/2023, established a benchmark for pre-implementation performance.

Subsequently, a comprehensive action plan was implemented, featuring daily educational sessions to reinforce I-PASS principles during the morning meetings by creating PowerPoint presentations (Microsoft Corporation, Redmond, WA, USA) to show the impact of proper handover.

Cycle two, or post-implementation, data was collected from 15/03/2023 to 28/03/2023, following a one-month observation period. By comparing pre- and post-implementation data to the I-PASS elements, the study sought to quantify the positive impact of the I-PASS protocol on handoff quality and patient safety.

During the data collection phase, observers attended handoff meetings in real-time. They were instructed to document whether each of the five I-PASS components was mentioned during the handoff by healthcare providers, using the Google Form checklist on a mobile device or computer. Observers were not blinded to the purpose of the study, as they were required to note adherence in real-time; however, they were trained to minimize bias in their recording.

Educational sessions

Following the conclusion of cycle one, an educational implementation was conducted from 02/02/2023 to 14/02/2023 across all Internal Medicine Units. Daily morning sessions (approximately 20-30 minutes each on weekdays) were led by the audit team in collaboration with the Internal Medicine Department. The sessions reviewed the rationale for structured handoffs, introduced the I-PASS components using locally adapted examples, and included brief demonstrations of good and suboptimal handoffs. All consultants, registrars, medical officers, and house officers were invited to attend; most staff who subsequently participated in handoffs during cycle two had attended at least one session, although detailed attendance logs were not collected. To reinforce implementation, I-PASS summary slides and printed reminders were displayed during morning meetings throughout the observation period.

Led by the audit team in collaboration with the Internal Medicine Department, observers involved in the study were thoroughly trained to ensure consistency in data collection and accuracy in documenting adherence to I-PASS. The training program included an overview of the I-PASS framework, a review of the checklist tool, and an interactive practical training session where observers practiced using the checklist with mock handoff video recordings. This practice ensured that all observers understood how to identify each component of I-PASS in real clinical handoffs.

Statistical analysis

Adherence to the I-PASS handoff protocol was measured using an Excel-based data collection tool. The collected data were subsequently analyzed using IBM SPSS Statistics for Windows, Version 26 (Released 2018; IBM Corp., Armonk, NY, USA). Each element of the I-PASS framework was categorized as either 'documented' or 'not documented.'

To assess the overall adherence rate, the frequency of documentation for each element was calculated as a percentage of total handoffs. Fisher’s exact test was used to assess differences in adherence proportions between cycles one and two, with two‑sided p‑values and a significance threshold of 0.05. Given the exploratory design and limited sample size, no multivariable analyses were performed, and potential confounding factors such as staff seniority, case complexity, and time of handoff were not controlled.

Data were collected using Google Forms, a freely available platform requiring no license. Statistical analyses were performed using IBM SPSS Statistics version 26. Microsoft Excel (Microsoft Corporation, Redmond, WA, USA) was used for initial data organization. The used tools were free and did not require a license or permission.

## Results

Cycle one of this study examined 55 patient handovers involving various critical conditions, while the second cycle assessed 50 handovers, resulting in a total of 105 handoff occurrences. A comprehensive list of the clinical cases included in each cycle is provided in Table 1.

**Table 1:** Clinical cases in both cycles

Cycle one (n =20)	Cycle two (n =18)
Multiple myeloma (1)	Hepatic encephalopathy (1)
Upper GI bleeding (2)	Pneumonia (2)
Intra-cerebral hemorrhage (1)	Lymphoma(1)
Ischemic stroke (1)	Megaloblastic anemia (1)
Hypertensive emergency (2)	Hemorrhagic stroke (3)
Sepsis (2)	Malaria (4)
Tuberculosis (2)	Sickle cell crisis (2)
Cerebral malaria (2)	Gullian-Barré syndrome (1)
Acute respiratory distress syndrome (1)	Diabetic ketoacidosis (1)
Acute kidney injury (2)	Lung cancer (1)
Sickle cell crisis (1)	Hepatic encephalopathy (1)
Gullian-Barré syndrome (1)	__
Severe malaria (1)	__
Acute pyelonephritis (1)	__

During cycle one, the study population comprised 20 patients. The gender distribution was skewed toward males, with a male-to-female ratio of 1.86:1. The patients' ages ranged from 20 to 90 years, with a mean age of 44.0 years and a standard deviation of 18.16 years.

In cycle one, adherence to the I-PASS handoff protocol showed variability. The "illness severity" component was properly documented in only 22/55 (40%) of handovers, while "patient summary" was captured in 37/55 (67%). The "action plan" had full compliance at 55/55 (100%); however, "situational awareness" lagged behind at just 20/55 (36%). Additionally, "synthesis by receiver" was noted in 37/55 (67%) of the handoffs (Table 2).

**Table 2:** Adequacy of completing the I-PASS form

I-PASS Item	Cycle 1 (n=55)	Cycle 2 (n=50)	OR (95% CI)	p-value
Illness Severity	22 (40%)	43 (86%)	9.82 (3.54–27.2)	<0.001
Patient Summary	37 (67%)	44 (88%)	3.56 (1.18–10.7)	0.019
Action Plan	55 (100%)	50 (100%)	Undefined	1.000
Situational Awareness	20 (36%)	42 (84%)	9.80 (3.59–26.7)	<0.001
Synthesis by Receiver	37 (67%)	48 (96%)	12.97 (2.84–59.2)	<0.001

Cycle two, which evaluated 50 handovers, included 18 patients. In this cycle, the gender distribution was more balanced, with a male-to-female ratio of 1:1. The patients' ages ranged from 20 to 66 years, with a mean age of 53.72 years and a standard deviation of 16.67 years.

Adherence improved markedly between cycles (Table 2). Illness severity increased from 22/55 (40%) to 43/50 (86%; p<0.001), and situational awareness from 20/55 (36%) to 42/50 (84%; p<0.001). Patient summary increased from 37/55 (67%) to 44/50 (88%, p=0.019), while action plan remained at 100% in both cycles. Synthesis by receiver improved from 37/55 (67%) to 48/50 (96%, p<0.001).

## Discussion

The significance of effective patient handoffs cannot be overstated, particularly in light of the alarming number of preventable medical errors. As highlighted by the Institute of Medicine's 2000 report, "To Err is Human," up to 98,000 individuals die annually in the United States due to these errors [18]. An ineffective handover, such as providing incomplete or incorrect information, can pose significant risks to both patient safety and staff well-being [19]. Given the high stakes involved, a standardized, structured approach to handoffs is essential.

I-PASS implementation in Sudan demonstrates multiple challenges. The health system in Sudan is deficient in proper data, divided information systems, clear policy, monitoring, and evaluation. Multiple challenges are faced by doctors because a shortage of supplies, lack of access, a low spending system, and poor referral systems all lead to challenges in effective changes [8].

For this study, the I-PASS mnemonic tool was implemented, a proven strategy for enhancing patient safety and reducing medical errors. Previous research has demonstrated I-PASS's effectiveness in decreasing medical errors by 23% and adverse events by 30% [20]. Additionally, studies have shown that I-PASS can be successfully applied in various clinical settings, including pediatric wards [21].

This study highlights the significant improvements observed in each element of the I-PASS handoff framework: illness severity, patient summary, action list, situational awareness and contingency planning, and synthesis by the receiver following structured training sessions and an emphasis on their importance. Another hallmark of this study would be the eye-opening need for wider implementation of such low-cost initiatives that would significantly affect the quality of care provided in Sudan and developing countries that face overwhelming challenges in their health systems. The impact of standardized handovers' effect on decreasing medical errors that are due to exhaustion and lack of manpower or resources is, therefore, greatly underestimated.

Training sessions enhanced the ability of healthcare providers to consistently assess and communicate illness severity, in which a pulse oximeter was used to assist us, ensuring that critical cases were prioritized appropriately, which showed a significant improvement of 40% pre-intervention vs. 86% post-intervention (p=0.0001) compared with a study done in the United States on I-PASS handoff in a pediatrics care unit in 2011-2012, which revealed (37% pre-intervention vs. 67% post-intervention, p=0.001) [3]. The structured approach also improved the patient summary by (67% pre-intervention vs 88% post-intervention, p=0.019), which aligns with another study conducted in a tertiary care pediatric hospital (73% pre-intervention vs 100% post-intervention, p=0.001) [21], which enhances a clearer and more complete information transfer, reducing the likelihood of omissions or unclear data.

The action list showed a consistent outcome (100% pre-intervention vs. 100% post-intervention, p=1) as the participants consider it the most critical step in the handover process. Whereas the study in the pediatrics care unit showed dramatic improvement (35% pre-intervention vs. 100% post-intervention, p < 0.001). Additionally, training strengthened situational awareness and contingency planning (36% pre-intervention vs. 84% post-intervention, p=0.0001), which aligns with the study in a tertiary care pediatrics hospital (16% pre-intervention vs. 73% post-intervention, p>0.00 which led to increasing awareness of potential complications and reinforcing the importance of proactive contingency strategies to manage critical issues.

Finally, synthesis by the receiver (67% pre-intervention vs. 96% post-intervention, p=0.0001), which benefited from encouraging active engagement from the receiving provider, leading to improved understanding and a reduction in miscommunication. However, surprisingly, in contrast with another study conducted in a tertiary care pediatric hospital, which showed a decrease in adherence to this element (11% pre-intervention vs. 9% post-intervention, p=0.001), they claim the reason is to avoid repeated information [21].

Overall, these improvements demonstrate the value of structured training in optimizing the I-PASS framework and enhancing patient safety during transitions of care.

Education is a crucial factor in enhancing the implementation of I-PASS. A study revealed that only 2/30 physicians had received specific education on handover. However, a notable improvement was observed following the implementation of an audit [22]. This trend is more pronounced in developing countries, where there is often a lower awareness of handover tools and their implementation may be more challenging due to limited education and lower baseline percentages [1].

Furthermore, a study suggested that individual physicians may have varying perceptions regarding the relevance of each mnemonic within I-PASS [22]. This underscores the importance of providing comprehensive education to highlight the significance of each tool and its contribution to effective handover.

Another study emphasized the critical role of education in I-PASS implementation. By training all direct caregivers and emphasizing the connection between higher-quality care and improved handover, the study successfully facilitated better adoption of I-PASS [23]. This finding aligns with the results of our own study.

Compared to other handover tools like SBAR, I-PASS has been shown to be more effective in improving communication and reducing the risk of errors [7,8,16]. Healthcare providers who have used I-PASS have reported increased confidence in their handover abilities and a better understanding of patient needs [8,9,24].

A key advantage of I-PASS is its efficiency. Studies have found that implementing I-PASS does not significantly increase the time required for handoffs compared to traditional methods [5]. While there may be an initial learning curve, the long-term benefits of improved patient safety and reduced medical errors outweigh the short-term costs [5].

Effective leadership and training are crucial for successful I-PASS implementation. A dedicated team of project managers can facilitate training and provide ongoing support to healthcare providers [23]. Additionally, integrating I-PASS into the electronic health record can enhance its usability and effectiveness.

Practical implementation lessons

Several implementation lessons emerged that may be relevant for similar settings. First, brief daily reinforcement during morning meetings, combined with visual prompts, appeared to sustain attention to I-PASS elements and support behavior change despite high workload. Second, explicit endorsement by unit leadership, particularly the consultant and registrars, signaled that structured handoffs were a departmental priority and encouraged junior staff to adopt the tool. Finally, the existing supervision model, house officers presenting with oversight from medical officers and registrars, facilitated real-time clarification and may have contributed to improved synthesis by the receiver in the post-implementation phase.

Strength and limitations 

This study offers several unique contributions to the existing literature. While previous research has primarily focused on simulated and e-learning environments to evaluate I-PASS, our study assessed its practical application in a real-world clinical setting.

Similar to another study [25], our research encountered limitations related to the reporting of safety-related handoffs. These incidents were infrequently reported, possibly due to concerns about negative consequences or limited transparency in the reporting process. The relatively small sample size and single-center design may not adequately reflect broader clinical practice, thereby limiting the generalizability of the findings and highlighting the need for larger multicenter audits. In addition, the absence of a control group limits the ability to attribute observed improvements solely to the intervention. Observer presence during handoff assessments may also have introduced Hawthorne and observer bias. Formal inter-rater reliability testing was not performed, which may have affected the consistency of observations. The study did not collect data on adverse events or error rates; therefore, the impact of I-PASS implementation on patient safety outcomes could not be assessed. Furthermore, adherence was measured as a binary yes/no outcome without assessment of the quality or accuracy of communication, which may overestimate the adequacy of some handoff elements. Potential confounders such as clinician experience, patient acuity, staffing levels, and time of day were not controlled and may have influenced the observed improvements.

## Conclusions

The implementation of the I-PASS framework in a low-resource internal medicine unit in Sudan was feasible and was associated with meaningful improvements in short-term adherence to structured handoff elements following targeted education. These findings suggest that structured, low-cost communication tools can be adapted and adopted in resource-limited settings with appropriate institutional support and leadership endorsement. The study did not measure adverse events or error rates; therefore, no conclusions can be drawn about the direct impact of I-PASS on patient safety outcomes.

## Appendices

Appendix 1

I-PASS is a handover tool used in healthcare to guarantee proper communication between the staff. It consists of five important components that, when integrated together, the handover is considered complete, it is concise, and straight to the point, which ensures that all the important information regarding the patients is provided in a timely manner despite the stress and workload in hospitals.

**Table 3:** Components of the I-PASS handover framework

Component	Letter	Key Elements
Illness Severity	I	Stable; Watcher; Unstable
Patient Summary	P	Summary statement; events leading to admission; hospital course; ongoing assessment; contingency plan
Action List	A	To-do list, timelines, and ownership
Situational Awareness & Contingency Planning	S	Awareness of current clinical status; plan for anticipated changes or deterioration
Synthesis by Receiver	S	Receiver summarizes what was heard; asks questions; restates key actions and to-do items
